# Detection of Bacterial Infection in Melon Plants by Classification Methods Based on Imaging Data

**DOI:** 10.3389/fpls.2018.00164

**Published:** 2018-02-14

**Authors:** Mónica Pineda, María L. Pérez-Bueno, Matilde Barón

**Affiliations:** Department of Biochemistry and Molecular and Cell Biology of Plants, Estación Experimental del Zaidín, Spanish National Research Council, Granada, Spain

**Keywords:** bacterial infection, chlorophyll fluorescence imaging, *Cucumis melo*, *Dickeya dadantii*, machine learning, multicolor fluorescence imaging, phenotyping, thermography

## Abstract

The bacterium *Dickeya dadantii* is responsible of important economic losses in crop yield worldwide. In melon leaves, *D. dadantii* produced multiple necrotic spots surrounded by a chlorotic halo, followed by necrosis of the whole infiltrated area and chlorosis in the surrounding tissues. The extent of these symptoms, as well as the day of appearance, was dose-dependent. Several imaging techniques (variable chlorophyll fluorescence, multicolor fluorescence, and thermography) provided spatial and temporal information about alterations in the primary and secondary metabolism, as well as the stomatal activity in the infected leaves. Detection of diseased leaves was carried out by using machine learning on the numerical data provided by these imaging techniques. Mathematical algorithms based on data from infiltrated areas offered 96.5 to 99.1% accuracy when classifying them as mock vs. bacteria-infiltrated. These algorithms also showed a high performance of classification of whole leaves, providing accuracy values of up to 96%. Thus, the detection of disease on whole leaves by a model trained on infiltrated areas appears as a reliable method that could be scaled-up for use in plant breeding programs or precision agriculture.

## Introduction

Precision agriculture is a combination of cultural and farming practices aimed to minimize human impact on the environment, while improving the crop yield. In the last years, non-invasive imaging sensors at several levels (from bench to remote sensing scale) have been implemented in precision agriculture, in order to easily identify problematic areas, to follow their evolution, or to evaluate possible losses in crop yield. This topic has generated much scientific literature, especially in recent years, as widely reviewed by Fiorani et al. (2012), Martinelli et al. (2015), Sankaran et al. (2015), Mahlein (2016), and Pérez-Sanz et al. (2017). The combination of imaging devices providing information about physiological processes appears as a good approach to monitor the effect of stress on crops (Chaerle et al., 2009; Barón et al., 2016).

Plant diseases induced by pathogens are responsible for major economic losses in global agriculture. In particular, the necrotrophic bacteria *Dickeya dadantii* causes great economic losses in crops and ornamental plants worldwide (Reverchon et al., 2016). In this work, the experimental host-pathogen system melon-*D. dadantii* was monitored by thermography, MCFI, and Chl-FI.

Thermography provides images of leaf temperature, dependent on the transpiration rate, which is controlled by the stomatal aperture (Jones, 1999, 2004). On the other hand, MCFI is based on the excitation of plant tissue with long-wavelength ultraviolet radiation (320–400 nm), which results in four characteristic fluorescence emission bands, peaking in the blue (F440), green (F520), red (F680), and far red (F740) regions of the spectrum (Buschmann and Lichtenthaler, 1998). The blue and green fluorescence are mainly emitted by phenolic compounds produced by secondary metabolism (Cerovic et al., 1999). In contrast, F680 and F740 are emitted by chlorophyll *a* (Gitelson et al., 1998). In addition, images corresponding to fluorescence ratios F440/F520, F440/F680, F440/F740, F520/F680, F520/F740, and F680/F740 can be useful in the detection of biotic stress (Buschmann and Lichtenthaler, 1998; Cerovic et al., 1999; Buschmann et al., 2000). Finally, the photosynthetic activity can be analyzed by variable chlorophyll fluorescence kinetics. Indeed, Chl-FI has been widely used to study the spatial and temporal heterogeneity of leaf photosynthesis under biotic stress, as reviewed by Rolfe and Scholes (2010) and Barón et al. (2016). Some of the parameters derived from Chl-FI are related to photochemical processes. Thus, F_V_/F_M_ represents the maximum quantum efficiency of PSII; Φ_PSII_, is the effective quantum yield of PSII; and qP is the photochemical quenching. On the other hand, non-photochemical quenching (as NPQ or qN) is related to energy dissipation (Maxwell and Johnson, 2000). Other Chl-FI parameters, although with no physiological meaning, can also be good reporters of stress (Soukupová et al., 2003; Pineda et al., 2008, 2011).

Imaging techniques such as thermography and Chl-FI, are routinely applied in precision agriculture and plant phenotyping (Costa et al., 2013; Porcar-Castell et al., 2014; Mahlein, 2016). However, even though MCFI has long been used in basic research to study plant stress, the use of this technique on proximal sensing is currently limited to few commercial devices (Cerovic et al., 2012; Latouche et al., 2015). Unfortunately, these devices are non-imaging sensors, limiting their applicability in high-throughput plant phenotyping in the field.

Machine learning addresses the analysis of big data by discovering relationships between the target output and the input features. Models or algorithms try to learn dependencies between these features and, based on that make predictions on new data. Nowadays, machine learning is broadly used in many aspects of life, such as manufacturing, computer vision, cybersecurity, clinical decision-making, climate change and plant sciences (Jordan and Mitchell, 2015). Common algorithms used in life sciences are LRA, SVM, and ANN. LRA is of particularly relevant in this field since it can be used to estimate the probability of a dichotomous outcome (for example “healthy” vs. “diseased”) based on one or more predictors or independent variables (Hosmer et al., 2013). On the other hand, SVM represents samples as points in a high-dimensional feature space, and support vectors define a hyperplane in that space. New samples will be predicted to belong to a certain category based on which side of the hyperplane they fall on (Behmann et al., 2015). Finally, ANN is a model inspired by biological neural networks that learn from input and output data (Hill et al., 1994), which is particularly suitable for the interpretation of information from optical sensors (Behmann et al., 2015).

The combination of Chl-FI, MCFI and thermography, providing information about physiological processes, followed by the use of classifying models is a reliable method suitable for precision disease management and plant breeding programs. Here, the classification methods LRA, SVM and ANN, trained on data obtained from melon leaves by the three imaging techniques, predict new samples as for “healthy” (control plants) vs. “*D. dadantii*-infected”. The classifiers obtained by this approach, trained on data from infiltrated areas, proved to have a high performance on whole leaves affected in a small portion of their surface (approximately 5–7% of the total leaf area).

## Materials and Methods

### Plant Material

Seeds of *Cucumis melo* (melon) v. Rochet Panal (Semillas Fitó, Barcelona, Spain) were sterilized in a 20% bleach solution during 2 min, rinsed twice in sterile water during 2 min and allowed to germinate in darkness on sterile water-soaked filter papers at 24°C for 1 week. Seedlings were then placed in pots containing 1:1 v/v coconut fiber and soil. Plants were grown at 150 μmol m^-2^ s^-1^ photosynthetically active radiation under 16/8 h light/dark photoperiod at 22/18°C day/night temperature and 65% relative humidity.

### Bacterial Growth and Infection

*Dickeya dadantii* (formerly known as *Erwinia chrysanthemi*) strain 3937 was grown for 24 h at 28°C in LB (Luria-Bertani) plates containing 25 mg ml^-1^ rifampicin. Bacterial suspensions were prepared in 10 mM MgCl_2_ at low or high dose: 10^4^ (LD) or 10^6^ (HD) colony forming units per ml, respectively.

Three weeks old plants were inoculated on the second leaf. Four or six regions were infiltrated by pressing the bacterial suspension into the abaxial side of the leaf using the blunt end of a 1 ml syringe, according to Pérez-Bueno et al. (2016a). Mock-inoculated control plants were infiltrated with 10 mM MgCl_2_. The size of the sample leaves at the beginning of the experiment was 100–120 cm^2^. Since each infiltration area covered about 1 cm^2^, the total area being infiltrated was approximately 5% of the total leaf area.

For image analysis purposes, three regions of interest were defined for each inoculation site (Supplementary Figure S1): the infiltrated area (I, accurately outlined using a marker pen), the neighboring area (N), and a distant region away from the I area (D). Measurements were taken at 3 and 7 days post-inoculation (dpi). Four to six plants per treatment and experiment were analyzed, and three independent experiments were carried out.

### Leaf Thermography

Infrared images of plant leaves were taken in the growth chamber using a FLIR A305sc camera (FLIR Systems, Wilsonville, OR, United States) vertically positioned approximately 500 mm above the leaves, according to Pérez-Bueno et al. (2015). For each leaf measurement, 10 thermal images were collected in the elapse of 10 s and averaged. The consequent image analysis was carried out using FLIR R&D software version 3.4. Thermal images were displayed using a false color scale. For further numerical analysis, average temperature was obtained for the three regions of interest or for whole leaf. Images correspond to standard experiments.

### Multicolor Fluorescence Imaging

Multicolor fluorescence imaging was performed on the adaxial side of mock-control and *D. dadantii*-infected melon leaves using an Open FluorCam FC 800-O (Photon Systems Instruments, Brno, Czechia), according to Pérez-Bueno et al. (2015). Images of F440, F520, F680, and F740 were captured by the FluorCam software version 7.1.0.3. This software also applied a false color scale to the black and white images recorded and to those calculated for the ratios F440/F520, F440/F680, F440/F740, F520/F680, F520/F740, and F680/F740. Average values for all available parameters from the three regions of interest, as well as for whole leaf, were calculated for mock-control and infected leaves. Images correspond to standard experiments.

### Chlorophyll Fluorescence Imaging

Chlorophyll fluorescence kinetics were performed by an open FluorCam 700MF (Photon System Instruments), according to protocol 1 from Pineda et al. (2008). A false color scale was applied to the black and white images using FluorCam software version 5.0. Images corresponding to chlorophyll fluorescence transient values (F_0_, F_M_, F_P_, F_M_′, F_t_, and F_0_′) were collected. In addition, images for the variable chlorophyll fluorescence parameters F_V_, F_V_/F_M_, F_M_/F_0_, F_V_/F_0_, RFd, qP, Φ_PSII_, and non-photochemical quenching (as qN and NPQ) were calculated by the same software according to the equation reviewed by Maxwell and Johnson (2000). Means of the above mentioned parameters were obtained for the three regions of interest previously defined, and for whole leaf. Images correspond to standard experiments.

### Data Analysis

Average values and standard errors for each of the parameters obtained by the different imaging techniques were calculated by Microsoft Office Excel 2010 (Microsoft Corporation, Redmond, WA, United States) for each region of interest. Student’s *t*-tests were carried out using SigmaPlot 13.0v (Systat Software, Inc., Richmond, CA, United States).

### Machine Learning

LRAs, SVMs, and ANNs were fitted to numerical data obtained from bacteria or mock-infiltrated leaves (labeled as “infected” and “healthy,” respectively) using SPSS version 23.0 software with the Essentials for R package (IBM Corporation, Armonk, NY, United States). Among all available parameters obtained by three different imaging techniques already described, only 16 were found to show statistically significant differences between treatments. Those parameters were selected as predictors for feeding the classifiers: temperature, F440, F520, F680, F740, F440/F520, F440/F680, F440/F740, F520/F680, F520/F740, F_V_/F_M_, F_M_/F_0_, NPQ, Φ_PSII_, qP, and qN. The models were performed separately for the three regions of interest, combining the data from 3 to 7 dpi from all three experiments. Out of 358 the samples available, two thirds were randomly selected for fitting the models (*n* = 239 for LRA, *n* = 238 for SVM, *n* = 241 for ANN). The remaining samples (*n* = 119 for LRA, *n* = 120 for SVM, *n* = 117 for ANN) were used for validation of the model performance of classification.

The binary LRAs were fitted following a standard method. On the other hand, the applied non-linear SVMs had a degree 3 Gaussian radial basis function kernel with a γ = 0.0625 (1/number of independent variables), cost = 1, coef0 = 0, 𝜀 = 0.1 and ν = 0.5. Finally, the ANNs, with two classes (“healthy” and “infected”), were based on multilayer perceptron, and the learning heuristic applied was resilient back-propagation. The ANN offering the best performance had one hidden layer with six neurons; an initial λ and σ of 5 × 10^-7^ and 5 × 10^-5^, respectively; an interval center of 0; an interval offset of ±0.5; activation functions in input and output layers were hyperbolic tangent and Softmax, respectively; an automatic selection of maximum epochs and batch as a training criteria. A summary of the training characteristics of the best classifiers is shown on Supplementary Table S1.

The performance of classifiers is evaluated by several parameters: specificity and sensitivity (percentage of correctly identified healthy or infected plants, respectively); accuracy (sum of true healthy and infected over the sample size); and F_1_ score (harmonic mean of precision and sensitivity, where precision is the number of true healthy samples divided by the number of true and false healthy plants). Supplementary Tables S2A, S3A shows confusion matrices for the corresponding validations, and the calculated statistical measures of the performance of their classifications, respectively.

The performance of the models fitted for I areas was evaluated on all available samples of whole leaves. LRA, SVM, and ANN were validated for every dpi taken either together or separately. In each case sample size was different, as is shown in **Figure 5A**. The total sample size was 74. Confusion matrices for the corresponding validations and the calculated statistical measures of the performance of their classifications are listed in Supplementary Tables S2B, S3B, respectively.

## Results

### Symptoms Caused by *D. dadantii* in Melon Leaves

**FIGURE 1:**
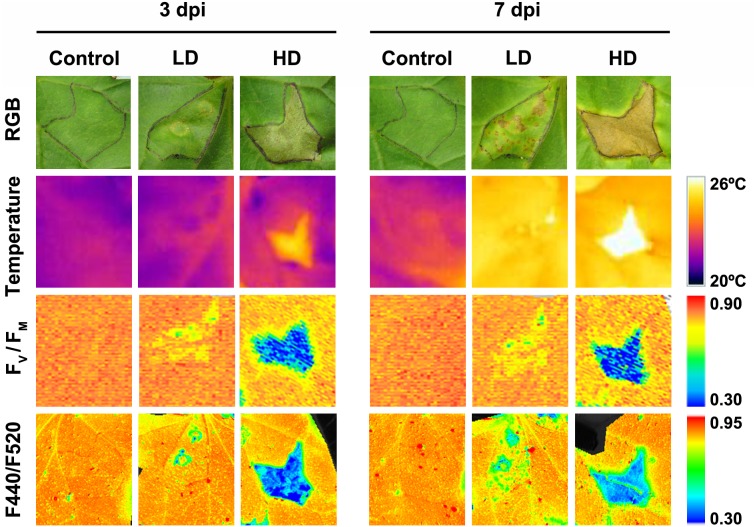
Evolution of symptoms caused by *Dickeya dadantii* in melon leaves inoculated at LD or HD (low and high dose, 10^4^ and 10^6^ colony forming units ml^-1^, respectively). The same mock-control and bacteria infiltrated areas are displayed for the RGB and leaf temperature images, as well as for the images calculated for F_V_/F_M_ and F440/F520 ratio. The corresponding color scale for each parameter is shown. F_V_/F_M_, maximum quantum efficiency of photosystem II; F440/F520, blue over green fluorescence; dpi, days post-inoculation.

Symptoms in the *D. dadantii*-inoculated melon leaves included chlorotic spots in areas infiltrated at LD at 3 dpi and necrotic spots surrounded by a chlorotic halo from 7 dpi (**Figure 1**). In the case of HD infected leaves, the I area appeared browned with multiple necrotic spots of very small diameter. At 7 dpi, the I tissues were dead and the N areas appeared chlorotic. No visible symptoms were found in the D areas of LD or HD inoculated leaves. From 7 dpi onward, there was no further evolution of the infection (data not shown). At this stage, the symptoms covered about 5–7% of the total leaf surface (in LD and HD infiltrated leaves, respectively). It is worth noticing here that control leaves did not develop any symptom related to the mock-infiltration along the period of study.

### Changes in *D. dadantii*-Infected Melon Leaves Metabolism

Leaf temperature was affected by *D. dadantii* to different extent depending upon bacterial dose (**Figure 1**). In LD infiltrated leaves at 3 dpi, only the I area displayed higher temperature than the corresponding mock-control; however, at 7 dpi, the whole leaf was warmer relative to the control. In the case of HD infiltration, the averaged temperature of whole leaves was higher for infected than for mock-control leaves. This effect was more severe at 7 dpi. This increment in temperature was higher in areas closer to the inoculation point.

**FIGURE 2:**
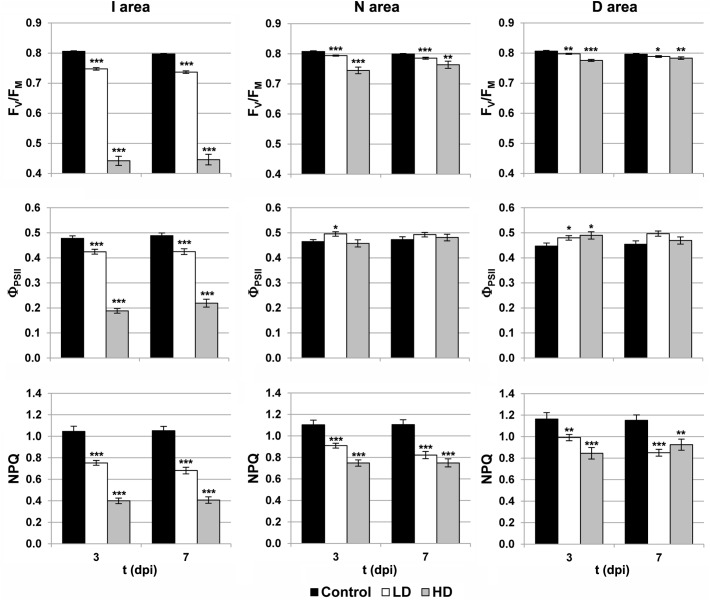
Parameters obtained by variable chlorophyll fluorescence imaging in the I (inoculated), N (neighboring), and D (distant) areas of treated leaves. F_V_/F_M_, maximum quantum efficiency of photosystem II; Φ_PSII_, effective quantum yield of photosystem II; NPQ, non-photochemical quenching; dpi, days post-inoculation; LD and HD, low and high bacterial dose (10^4^ and 10^6^ colony forming units ml^-1^, respectively). ^∗^*p* < 0.1, ^∗∗^*p* < 0.01, and ^∗∗∗^*p* < 0.001, according to Student’s *t*-test.

Photosynthesis is affected by *D. dadantii* infection, as shown by the parameters obtained from chlorophyll fluorescence kinetics (**Figures 1, 2**). The values of F_V_/F_M_ and NPQ were significantly lower relative to the corresponding areas of mock-control leaves. This effect was found in the three regions of interest at every dpi assayed, and was more severe upon HD infiltration. On the other hand, I areas of infected leaves showed lower Φ_PSII_ values relative to the control I areas. In the rest of the leaf, slightly higher values of this parameter were found only at 3 dpi in D areas of bacteria-infiltrated leaves (LD and HD), and also in N areas (only in the case of LD).

**FIGURE 3:**
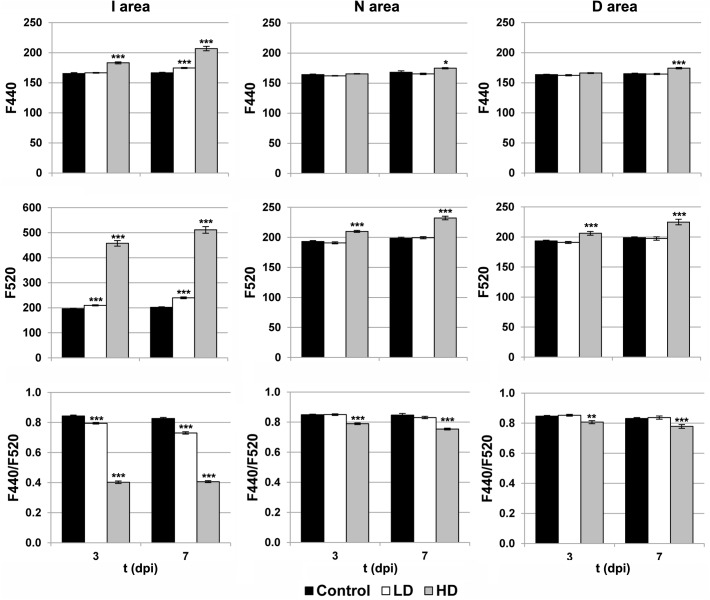
Parameters obtained by MCFI in the I (inoculated), N (neighboring), and D (distant) areas of treated leaves. F440, fluorescence emitted at 440 nm; F520, fluorescence emitted at 520 nm; dpi, days post-inoculation; LD and HD, low and high bacterial dose (10^4^ and 10^6^ colony forming units ml^-1^, respectively). ^∗^*p* < 0.1, ^∗∗^*p* < 0.01, and ^∗∗∗^*p* < 0.001, respectively, according to Student’s *t*-test.

The activity of the secondary metabolism was analyzed by MCFI (**Figures 1, 3**). In the case of HD inoculation, the whole leaf showed a drastic increase on F520 at 3 and 7 dpi. In contrast, F440 showed a moderate increase in I areas at 3 dpi and the rest of the leaf at 7 dpi, relative to the mock-control. On the other hand, the MCFI parameter F440/F520 was significantly decreased across HD inoculated leaves during the period of study. In the case of inoculation with LD, F440 (at 7 dpi) and F520 (at 3 and 7 dpi) showed a small but statistically significant increase but limited to the I area. Moreover, F440/F520 only decreased significantly in I areas in response to the infection.

### Machine Learning to Classify Leaf Areas as “Healthy” or “*D. dadantii*-Infected”

Among all available parameters obtained by thermography, MCFI and Chl-FI, only 16 showed statistically significant differences between control and infected plants in the three regions of interest, according to Student’s *t*-test (*p* < 0.001). These parameters (temperature, F440, F520, F680, F740, F440/F520, F440/F680, F440/F740, F520/F680, F520/F740, F_V_/F_M_, F_M_/F_0_, NPQ, Φ_PSII_, qP, and qN) were the input features for LRA, SVM, and ANN models to classify melon leaves as healthy or infected.

**FIGURE 4:**
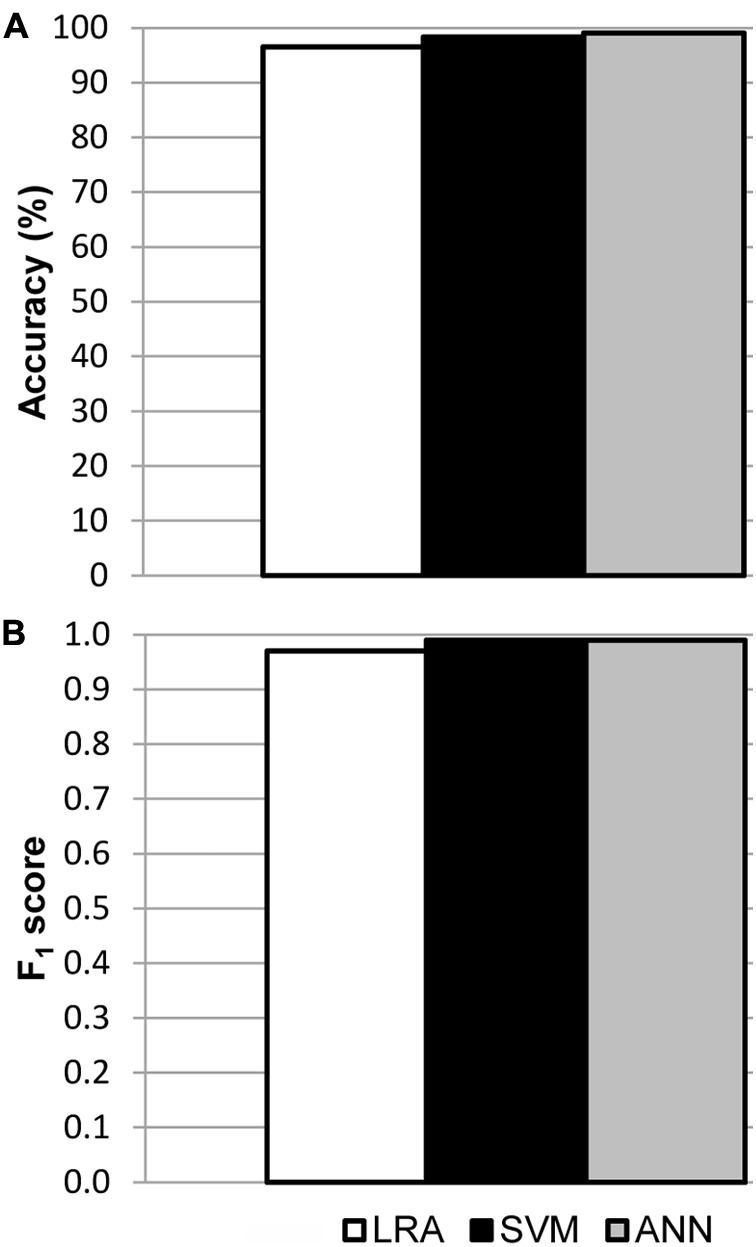
Results of the validation of the I area models used to classify healthy and *D. dadantii*-infected I areas. Classifiers were trained and tested combining data from the mock-control, LD, and HD infiltrated areas, at every day post-inoculation assayed. **(A)** Accuracy of the models. **(B)** F_1_ score of the classifiers. n_test_ = 119 for LRA, 120 for SVM, and 117 for ANN.

Models were fitted for every region of interest separately, using all available data from mock, LD and HD inoculated leaves at every dpi assayed in the three independent experiments. The best fits were those obtained for I areas, from now on I area models (Supplementary Table S1). In each case, the sample size for training and for testing the models is shown in Supplementary Table S2A, as well as the corresponding confusion matrices. The accuracy of the best LRA, SVM, and ANN was 96.5, 98.3, and 99.1%, respectively (**Figure 4A**), and their F_1_ score was 0.97, 0.99, and 0.99, respectively (**Figure 4B**). Values of specificity and sensitivity for every model are displayed in Supplementary Table S3A. The performance of the fitted models for N areas was much lower, with accuracy lower than 90%. On the contrary, no algorithm could be fitted for the data from D areas (data not shown). Therefore, only I areas provided valuable information that could be used by machine learning to obtain accurate classifying models.

### Machine Learning to Classify Whole Leaves as “Healthy” or “*D. dadantii*-Infected”

**FIGURE 5:**
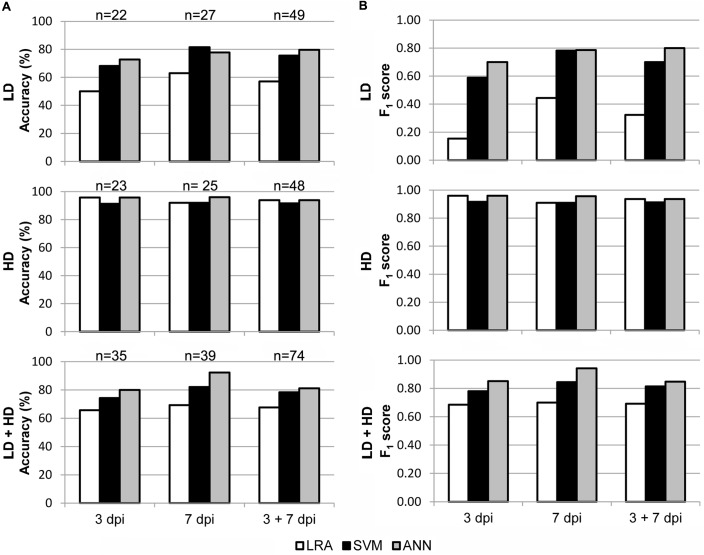
Results of the validation of the I area models used to classify whole leaves as healthy and *D. dadantii*-infected. Classifiers were trained with data from all available I areas and tested on the data set for whole leaves. **(A)** Accuracy of the models. **(B)** F_1_ score of the classifiers. dpi, days post-inoculation; LD and HD, low and high bacterial dose (10^4^ and 10^6^ colony forming units ml^-1^, respectively). n_test_ is specified in **(A)**.

In order to test the applicability of the I area models to entire leaves, a new set of data was generated by analyzing whole leaves. Then, the I area models were validated with the data from healthy vs. LD and HD infected leaves at 3 and 7 dpi, separately or together. Furthermore, to better reproduce a more realistic scenario, the models were also tested with all the data from every treatment at every dpi assayed. The accuracy and F_1_ score of I area models (LRA, SVM, and ANN) applied to data obtained from whole leaves, as well as the sample size for the validation, are shown in **Figure 5**. Confusion matrices are shown on Supplementary Table S2B and the statistical parameters of the validations on Supplementary Table S3B.

The three models, especially the LRA and the ANN, were most accurate when classifying HD infiltrated vs. mock-control leaves. In that case, F_1_ score ranged from 0.91 to 0.96 when classifying samples at 3 dpi and 7 dpi separately or together. On the contrary, the ANN provided the best performance when identifying LD infiltrated leaves vs. healthy plants. Thus, the F_1_ score was 0.79–0.80 when samples at 7 dpi alone, or 3 + 7 dpi, were considered. However, SVM showed a lower performance, and the LRA was not able to classify LD infiltrated vs. healthy leaves. Moreover, the ANN outperformed the other two models when classifying LD and HD infiltrated together vs. healthy leaves (F_1_ score of 0.85, 0.94, and 0.85 when considering 3, 7, or 3 + 7 dpi samples, respectively).

## Discussion

### Effect of *D. dadantii* Infection on Melon Plant Metabolism

*Dickeya dadantii* infected melon plants showed alterations in metabolism monitored by imaging sensors. An enhancement of the secondary metabolism could be responsible for the increase in blue and green fluorescence in N and D areas of HD leaves, emitted mainly by phenolic compounds. Many of them, such as precursors of lignin or phytoalexins among others, could play important roles in plant defense against *D. dadantii* (Cerovic et al., 1999; Mazid et al., 2011). It is worth noticing here that in the particular case of I areas, changes in the optical properties of the tissue, due to loss in water content, would also contribute to the increase in F440 and F520 (Cerovic et al., 1999; Takács et al., 2000).

On the other hand, a drastic increase in temperature of whole leaves in response to the infection is indicative of activation of stomatal closure, which decreases the transpiration rate across those leaves. Stomatal closure, triggered by the detection of pathogen associated molecular patterns, is part of a plant innate immune response to restrict pathogen entry (Melotto et al., 2008; Zeng et al., 2010).

Concomitantly with the enhancement of secondary metabolism and the induction of stomatal closure, photosynthesis was impaired in the infected plants. The extent of this impairment also correlated positively with the pathogen dose. The bacteria-infiltrated areas showed a decrease in F_V_/F_M_, Φ_PSII_, and also in NPQ, suggesting a loss in functionality of the thylakoid and a severe decrease in the photosynthetic activity, particularly in the HD leaves. This effect preceded the development of necrosis in the infiltrated tissue. Bacterial challenge has been previously shown to cause a decrease in photosynthesis in the inoculation sites, as part of the plant defense program to limit carbon source availability for pathogens and/or to redirect carbon into secondary metabolism (Bolton, 2009; Barón et al., 2016).

On the contrary, the photosynthetic activity in N areas of LD infected leaves and D areas of LD and HD inoculated leaves appeared to be enhanced at 3 dpi, relative to the mock-control. Increase of photosynthesis rate in uninfected tissues surrounding the infection site is characteristic of some virulent pathogens (Chou et al., 2000; Berger et al., 2004; Rojas et al., 2014). This increment in the efficiency of PSII could contribute to cover the great metabolic demand of energy necessary to trigger the defense response (Bolton, 2009; Murchie and Lawson, 2013).

### Use of Mathematical Tools to Identify Infiltration Sites on Melon Leaves as “Healthy” or “*D. dadantii*-Infected”

The data obtained by thermography, Chl-FI, and MCFI were used to feed classifying algorithms for each region of interest. The high accuracy and F_1_ score of the I area models (LRA, SVM, and ANN) when classifying such area are comparable to those obtained by other authors. Berdugo et al. (2014) applied a general linear model to classify cucumber leaves fully infected by either fungal or viral pathogens, and obtained accuracies from 85 to 100%. On the other hand, tomato leaves infected with the fungus *Oidium neolycopersici* were classified with 60–90% accuracy (Raza et al., 2015). The same host plant species infected with the fungus *Phytophthora infestans* could be classified with an accuracy up to 100% using extreme learning machine (Xie et al., 2015). A method based on radial-basis function network classified resistant and tolerant cultivars of sugar beet infected with *Cercospora beticola* with accuracies ranging from 69.4 to 99.9% (Arens et al., 2016). In contrast with the study here presented, these works considered leaves or plants completely invaded by the pathogen, corresponding to a very advanced stage of the infection.

### Use of Mathematical Tools to Classify Whole Leaves as “Healthy” or “*D. dadantii*-Infected”

In a similar study, ANNs and LRAs were fitted to numerical data obtained by MCFI and thermography from regions of interest in zucchini leaves infected by *D. dadantii* (Pérez-Bueno et al., 2016b). These classifiers were only valid for the corresponding areas of zucchini and melon leaves. The applicability of models based on symptomatic areas relies on a prior identification of areas presenting visual symptoms. Moreover, even when this recognition process could be automated using computer vision (Camargo and Smith, 2009; Raza et al., 2015), the whole method of diagnosis could presumably be more time consuming and less reliable, particularly in the case of weak symptoms or early phases of the infection. Therefore, a model valid for whole leaves or plants, even at early stages of the infection, when symptoms were weak and/or covered a small portion of the tissues, would be more easily implemented. In contrast to Pérez-Bueno et al. (2016b), the models presented here (ANN, LRA as well as SVM) showed a very high performance when classifying, not only I areas, but most importantly when classifying whole leaves. This is due to the use of Chl-FI parameters as predictors together with those from MCFI and thermography.

Achieving high sensitivity and therefore low false negatives rate is most important when detecting diseased plants (Sankaran et al., 2013), in this particular case *D. dadantii*-infected classified as healthy. Having this into account, ANN outperformed the other two models when classifying whole leaves. Moreover, this algorithm provided the best accuracy and F_1_ score when classifying the complete dataset for whole leaves (3 + 7 dpi of LD and HD vs. control), followed by the SVM. Both ANN and SVM are particularly suitable for the interpretation of data from optical sensors because the included noise factors can be compensated by a sufficient amount of representative training data (Behmann et al., 2015). Nonetheless, ANN is currently applied in many complex real-world problems. The use of ANN in the field of image processing and remote sensing has increased rapidly because of the ability of this algorithm to handle large volume of complex data for processing and classification (Hahn, 2009). On the other hand, LRA is a very flexible and robust method, but requires many more data to achieve stable, meaningful results (Hosmer et al., 2013). This could explain why the LRA provided the poorest results when classifying whole leaves.

Only few works have addressed the classification of plants with patchy symptoms by training models on data obtained from whole leaves or plants. For example, Calderón et al. (2015) analyzed Verticillum wilt severity in olive trees by high-resolution thermal and hyperspectral imagery in a crop field. When classifying initial and low severity levels, a linear discriminant analysis reached an accuracy of 71.4 and 75.0%, respectively. Whole leaves of oilseed rape partly infected with *Alternaria* were analyzed by thermal and hyperspectral imaging providing an ANN model with 90% accuracy (Baranowski et al., 2015). The degree of severity of symptoms produced by the grapevine leafroll disease in entire trees could be detected with an accuracy of 75–94.4% when using an ant colony clustering algorithm to multispectral imaging data (Hou et al., 2016). Similarly, SVM applied to data derived from thermography and reflectance images, provided an accuracy of about 87% when classifying citrus presenting a few huanglongbing symptomatic leaves (Sankaran et al., 2013). On the other hand, algorithms based on parameter derived from F520/F680 histograms from whole leaves were used for the identification of infected zucchini leaves (Pineda et al., 2017). In that case, an ANN rendered the highest accuracy when identifying zucchini leaves infected by bacteria or fungus. In contrast to these works, the use of MCFI, thermography, and Chl-FI to fit models for the I areas improved the performance of such models for the classification of whole leaves. Particularly, the ANN showed similar or even better performance than those models discussed above. This result is especially relevant considering that only 5–7% of the total leaf surface was affected by infection. Consequently, this strategy could be especially suitable for application on plant phenotyping or precision agriculture.

## Author Contributions

MP and MP-B took part in the experimental design, acquisition, analysis, and interpretation of data, and on writing up. MB took part in the experimental design, the interpretation of data and writing up.

## Conflict of Interest Statement

The authors declare that the research was conducted in the absence of any commercial or financial relationships that could be construed as a potential conflict of interest.

## Abbreviations

ANN: artificial neural network
Chl-FI: variable chlorophyll fluorescence imaging
D: distant
dpi: days post-inoculation
F440: blue fluorescence
F520: green fluorescence
F680: red fluorescence
F740: far red fluorescence
F_V_/F_M_: maximum quantum efficiency of photosystem II
HD: high bacterial dose
I: infiltrated
LD: low bacterial dose
LRA: logistic regression analysis
MCFI: multicolor fluorescence imaging
N: neighboring
NPQ: non-photochemical quenching
PSII: photosystem II
Φ_PSII_: effective quantum yield of photosystem II
qN: non-photochemical quenching
qP: photochemical quenching
SVM: support vector machine
