# Supplementary material for: Detection of Bacterial Infection in Melon Plants by Classification Methods Based on Imaging Data
Source: Front Plant Sci. 2018 Feb 14;9:164. doi: 10.3389/fpls.2018.00164 (PMC5817087; doi:10.3389/fpls.2018.00164)
Supplement: Supplementary file 1 [file Table_1.docx]

Supplementary Table 1. Summary of the characteristics of the classifiers trained on data from the infiltrated area.

LRA (Logistic regression analysis): Summary of characteristics of the best LRA trained with data of I areas from healthy and *D. dadantii*-infected melon leaves. n=239. In all cases, degrees of freedom=1. B: intercept (coefficient for the constant in the null model). C.I.: Confidence interval: 95%. Other features of the LRA are: cut: 0.5; maximum iterates: 20.

SVM (support vector machine): Summary of characteristics of the best SVM model trained with data of I areas from healthy and *D. dadantii*-infected melon leaves. n=238. Number of support vectors: 72. Other features of the SVM are: type: c classification; radial Kernel; Kernel degree and coef0: ND; γ: 0.0625; degree: 3; coef0: 0; cost of the wrong classification: 1; ε: 0.1; ν: 0.5.

ANN (artificial neural network): Summary of characteristics of the best multilayer perceptron ANN model trained with data of I areas from healthy and *D. dadantii*-infected melon leaves. The learning heuristic applied was resilient backpropagation. n=241. This ANN had one hidden layer with six neurons and two classes (“control” vs. “infected”). Cross-entropy error of training and testing batches: 12.25 and 3.362, respectively. Other features of the ANN are: Initial λ: 5·10^-7^; initial σ: 5·10^-5^; interval center: 0; interval offset: ±0.5; activation functions in input and output layers: hyperbolic tangent and Softmax, respectively; automatic selection of maximum epochs; training criteria: batch.

F440: blue fluorescence. F520: green fluorescence. F680: red fluorescence. F740: far red fluorescence. F_V_/F_M_: maximum quantum yield of photosystem II. Φ_PSII_: quantum yield of photosystem II. qP: photochemical quenching. NPQ and qN: non-photochemical quenching.

| **LRA** | | | | | | | | |
| --- | --- | --- | --- | --- | --- | --- | --- | --- |
| **Variables** | **B** | **Standard error** | **Wald chi^2^** | ***p* value** | | **Exp(B)** | **95% C.I. for Exp(B)** | |
|  |  |  |  |  |  |  | **Lower limit** | **Upper limit** |
| Temperature | -1.815 | 0.874 | 4.315 | 0.038 | | 0.163 | 0.029 | 0.903 |
| F440 | 0.870 | 0.638 | 1.858 | 0.173 | | 2.386 | 0.683 | 8.332 |
| F520 | -0.270 | 0.324 | 0.691 | 0.406 | | 0.764 | 0.404 | 1.442 |
| F680 | -0.186 | 0.223 | 0.700 | 0.403 | | 0.830 | 0.537 | 1.284 |
| F740 | 0.038 | 0.089 | 0.183 | 0.669 | | 1.039 | 0.872 | 1.238 |
| F440/F520 | -121.659 | 62.599 | 3.777 | 0.052 | | 0.000 | 0.000 | 2.811 |
| F440/F680 | -92.482 | 208.542 | 0.197 | 0.657 | | 0.000 | 0.000 | 2.223·10^137^ |
| F440/F740 | 14.205 | 205.553 | 0.005 | 0.945 | | 1476231.047 | 0.000 | 1.370·10^181^ |
| F520/F680 | -129.673 | 121.148 | 1.146 | 0.284 | | 0.000 | 0.000 | 6.382·10^46^ |
| F520/F740 | 180.043 | 181.523 | 0.984 | 0.321 | | 1.555·10^78^ | 0.000 | 5.062·10^232^ |
| F_V_/F_M_ | -233.292 | 238.814 | 0.954 | 0.329 | | 0.000 | 0.000 | 9.161·10^101^ |
| F_M_/F_0_ | 11.308 | 12.437 | 0.827 | 0.363 | | 81468.672 | 0.000 | 3142·10^15^ |
| Φ_PSII_ | -78.083 | 30.673 | 6.480 | 0.011 | | 0.000 | 0.000 | 0.000 |
| qP | -41.020 | 25.183 | 2.653 | 0.103 | | 0.000 | 0.000 | 4175.936 |
| NPQ | -24.752 | 38.979 | 0.403 | 0.525 | | 0.000 | 0.000 | 2.68·10^22^ |
| qN | -17.808 | 97.351 | 0.033 | 0.855 | | 0.000 | 0.000 | 1.355·10^75^ |
| Constant | 391.178 | 245.885 | 2.531 | 0.112 | | 7.700·10^169^ | - | - |
| **SVM** | | | | | | | | |
| **Independent Variables** | **Variable scaling** | | **Feature weights** | **Independent Variables** | | **Variable scaling** | | **Feature weights** |
|  | **Centre** | **Scale** |  |  |  | **Centre** | **Scale** |  |
| Temperature | 23.699 | 1.540 | 1.243 | F520/F680 | | 0.721 | 0.367 | -4.247 |
| F440 | 177.137 | 20.143 | -5.332 | F520/F740 | | 0.482 | 0.332 | -4.629 |
| F520 | 285.467 | 132.129 | -3.986 | F_V_/F_M_ | 0.661 | | 0.160 | 5.895 |
| F680 | 412.238 | 93.586 | 3.374 | F_M_/F_0_ | 3.538 | | 1.304 | 10.298 |
| F740 | 673.191 | 194.791 | 8.520 | Φ_PSII_ | 0.367 | | 0.122 | 11.768 |
| F440/F520 | 0.697 | 0.172 | 5.199 | qP | 1.103 | | 0.162 | -0.869 |
| F440/F680 | 0.447 | 0.095 | -5.297 | NPQ | 0.710 | | 0.316 | 9.921 |
| F440/F740 | 0.287 | 0.101 | -6.538 | qN | 0.597 | | 0.099 | 4.439 |

| **ANN** | | | | | | | |
| --- | --- | --- | --- | --- | --- | --- | --- |
|  | **Parameter estimates** | | | | | | **Normalized**  **Importance (%)** |
| **Independent**  **variable** | **Neuron 1**  **in hidden layer** | **Neuron 2**  **in hidden layer** | **Neuron 3**  **in hidden layer** | **Neuron 4**  **in hidden layer** | **Neuron 5**  **in hidden layer** | **Neuron 6**  **in hidden layer** |  |
| Temperature | 0.101 | 0.441 | -0.060 | -0.313 | -0.111 | -0.429 | 47.2 |
| F440 | -0.406 | 0.243 | 0.398 | 0.160 | -0.129 | 1.238 | 90.0 |
| F520 | -0.138 | 0.338 | -0.445 | 0.237 | 0.031 | -0.469 | 25.3 |
| F680 | -0.320 | 0.590 | 0.343 | -0.342 | 0.501 | 0.702 | 35.8 |
| F740 | -0.130 | 0.428 | -0.302 | 0.445 | -0.042 | -0.792 | 61.2 |
| F440/F520 | 0.244 | 0.259 | -0.005 | -0.232 | 0.061 | -0.114 | 14.4 |
| F440/F680 | 0.019 | 0-.463 | 0.187 | -0.037 | -0.283 | 0.184 | 21.2 |
| F440/F740 | 0.334 | -0.585 | 0.077 | 0.237 | -0.303 | 0.305 | 50.4 |
| F520/F680 | -0.322 | -0.410 | 0.074 | -0.502 | -0.145 | -0.366 | 13.9 |
| F520/F740 | 0.513 | -0.296 | 0.025 | -0.358 | 0.211 | -0.001 | 20.1 |
| F_V_/F_M_ | 0.348 | 0.361 | 0.301 | 0.932 | -0.313 | 0.395 | 16.9 |
| F_M_/F_0_ | -0.012 | -0.047 | -0.962 | -0.010 | 0.181 | -1.016 | 58.8 |
| Φ_PSII_ | 0.341 | -0.319 | -0.416 | -0.339 | -0.126 | -1.555 | 100.0 |
| qP | 0.360 | -0.153 | 0.253 | 0.045 | 0.314 | -0.665 | 86.4 |
| NPQ | -0.073 | -0.324 | -0.358 | 0.229 | 0.396 | -1.145 | 85.6 |
| qN | 0.275 | 0.330 | -0.390 | 0.002 | 0.037 | -0.623 | 70.8 |
| Bias | 0.491 | -0.300 | 1.049 | -0.166 | 0.200 | 2.397 | - |
