# Supplementary material for: Detection of Bacterial Infection in Melon Plants by Classification Methods Based on Imaging Data
Source: Front Plant Sci. 2018 Feb 14;9:164. doi: 10.3389/fpls.2018.00164 (PMC5817087; doi:10.3389/fpls.2018.00164)
Supplement: Supplementary file 2 [file Table_2.DOCX]

**Supplementary Tables 2**. Confusion matrices for the validation of classifying algorithms. I area models algorithms were trained and tested with data from I areas (2.A). These models were also tested with data from whole leaves (2.B). LRA, logistic regression analysis; SVM, support vector machine; ANN, artificial neural network; n, sample size. In the table 2.B, sample size for training the models was 358 in all cases. Sample size for testing is the sum of all samples contained in the corresponding confusion matrix.

Suppl. Table 2.A

| **LRA** | **Predicted** | | **n** | |
| --- | --- | --- | --- | --- |
| **Observed** | **Control** | **Infected** | **Training** | **Test** |
| **Control** | 36 | 2 | 239 | 119 |
| **Infected** | 2 | 79 |  |  |
| **SVM** | **Predicted** | | **n** | |
| **Observed** | **Control** | **Infected** | **Training** | **Test** |
| **Control** | 36 | 1 | 238 | 120 |
| **Infected** | 1 | 82 |  |  |
| **ANN** | **Predicted** | | **n** | |
| **Observed** | **Control** | **Infected** | **Training** | **Test** |
| **Control** | 35 | 0 | 241 | 117 |
| **Infected** | 1 | 81 |  |  |

Suppl. Table 2.B

| **LRA** | | | | | | | | | |
| --- | --- | --- | --- | --- | --- | --- | --- | --- | --- |
| **LD** | **3 dpi** | Predicted | | **7 dpi** | Predicted | | **3 + 7 dpi** | Predicted | |
|  | Observed | Control | Infected | Observed | Control | Infected | Observed | Control | Infected |
|  | Control | 10 | 0 | Control | 13 | 0 | Control | 23 | 0 |
|  | Infected | 11 | 1 | Infected | 10 | 4 | Infected | 21 | 5 |
| **HD** | **3 dpi** | Predicted | | **7 dpi** | Predicted | | **3 + 7 dpi** | Predicted | |
|  | Observed | Control | Infected | Observed | Control | Infected | Observed | Control | Infected |
|  | Control | 10 | 0 | Control | 13 | 0 | Control | 23 | 0 |
|  | Infected | 1 | 12 | Infected | 2 | 10 | Infected | 3 | 22 |
| **LD + HD** | **3 dpi** | Predicted | | **7 dpi** | Predicted | | **3 + 7 dpi** | Predicted | |
|  | Observed | Control | Infected | Observed | Control | Infected | Observed | Control | Infected |
|  | Control | 10 | 0 | Control | 13 | 0 | Control | 23 | 0 |
|  | Infected | 12 | 13 | Infected | 12 | 14 | Infected | 24 | 27 |
| **SVM** | | | | | | | | | |
| **LD** | **3 dpi** | Predicted | | **7 dpi** | Predicted | | **3 + 7 dpi** | Predicted | |
|  | Observed | Control | Infected | Observed | Control | Infected | Observed | Control | Infected |
|  | Control | 10 | 0 | Control | 13 | 0 | Control | 23 | 0 |
|  | Infected | 7 | 5 | Infected | 5 | 9 | Infected | 12 | 14 |
| **HD** | **3 dpi** | Predicted | | **7 dpi** | Predicted | | **3 + 7 dpi** | Predicted | |
|  | Observed | Control | Infected | Observed | Control | Infected | Observed | Control | Infected |
|  | Control | 10 | 0 | Control | 13 | 0 | Control | 23 | 0 |
|  | Infected | 2 | 11 | Infected | 2 | 10 | Infected | 4 | 21 |
| **LD + HD** | **3 dpi** | Predicted | | **7 dpi** | Predicted | | **3 + 7 dpi** | Predicted | |
|  | Observed | Control | Infected | Observed | Control | Infected | Observed | Control | Infected |
|  | Control | 10 | 0 | Control | 13 | 0 | Control | 23 | 0 |
|  | Infected | 9 | 16 | Infected | 7 | 19 | Infected | 16 | 35 |
| **ANN** | | | | | | | | | |
| **LD** | **3 dpi** | Predicted | | **7 dpi** | Predicted | | **3 + 7 dpi** | Predicted | |
|  | Observed | Control | Infected | Observed | Control | Infected | Observed | Control | Infected |
|  | Control | 9 | 1 | Control | 10 | 3 | Control | 19 | 4 |
|  | Infected | 5 | 7 | Infected | 3 | 11 | Infected | 6 | 20 |
| **HD** | **3 dpi** | Predicted | | **7 dpi** | Predicted | | **3 + 7 dpi** | Predicted | |
|  | Observed | Control | Infected | Observed | Control | Infected | Observed | Control | Infected |
|  | Control | 10 | 0 | Control | 13 | 0 | Control | 23 | 0 |
|  | Infected | 1 | 12 | Infected | 1 | 11 | Infected | 3 | 22 |
| **LD + HD** | **3 dpi** | Predicted | | **7 dpi** | Predicted | | **3 + 7 dpi** | Predicted | |
|  | Observed | Control | Infected | Observed | Control | Infected | Observed | Control | Infected |
|  | Control | 8 | 2 | Control | 12 | 1 | Control | 21 | 2 |
|  | Infected | 5 | 20 | Infected | 2 | 24 | Infected | 12 | 39 |
