# Supplementary material for: Detection of Bacterial Infection in Melon Plants by Classification Methods Based on Imaging Data
Source: Front Plant Sci. 2018 Feb 14;9:164. doi: 10.3389/fpls.2018.00164 (PMC5817087; doi:10.3389/fpls.2018.00164)
Supplement: Supplementary file 3 [file Table_3.DOCX]

**Supplementary Tables 3. Classification performance of the three models.** Classification performance of the logistic regression analysis (LRA), support vector machine (SVM) and artificial neural network (ANN). The I area models were trained and tested with the data from I areas (3.A). These models were also tested with the data set from whole leaves (3.B). dpi, days post-inoculation; LD and HD, low and high bacterial-dose (10^4^ and 10^6^ colony forming units ml^-1^, respectively); n_training_, sample size for training; n_test_, sample size for validation.

Suppl. Table 3.A

|  | **LRA** | **SVM** | **ANN** |
| --- | --- | --- | --- |
| **Specificity (%)** | 94.7 | 97.3 | 100 |
| **Sensitivity (%)** | 97.3 | 98.8 | 98.8 |
| **Accuracy (%)** | 96.5 | 98.3 | 99.1 |
| **F_1_ score** | 0.97 | 0.99 | 0.99 |
| **n_training_** | 238 | 238 | 241 |
| **n_test_** | 120 | 120 | 117 |

Suppl. Table 3.B

|  | | **LD** | | | **HD** | | | **LD + HD** | | |
| --- | --- | --- | --- | --- | --- | --- | --- | --- | --- | --- |
|  |  | 3 dpi | 7 dpi | 3 + 7 dpi | 3 dpi | 7 dpi | 3 + 7 dpi | 3 dpi | 7 dpi | 3 + 7 dpi |
| **Specificity (%)** | LRA | 100 | 100 | 100 | 100 | 100 | 100 | 100 | 100 | 100 |
|  | SVM | 100 | 100 | 100 | 100 | 100 | 100 | 100 | 100 | 100 |
|  | ANN | 90.0 | 76.9 | 82.6 | 100 | 100 | 100 | 80.0 | 92.3 | 91.3 |
| **Sensitivity (%)** | LRA | 8.3 | 28.6 | 19.2 | 92.3 | 83.3 | 88.0 | 52.0 | 53.8 | 52.9 |
|  | SVM | 41.7 | 64.3 | 53.8 | 84.6 | 83.3 | 84.0 | 64.0 | 73.1 | 68.6 |
|  | ANN | 58.3 | 78.6 | 76.9 | 92.3 | 91.7 | 88.0 | 80.0 | 92.3 | 76.5 |
| **Accuracy (%)** | LRA | 50 | 63 | 57.1 | 95.7 | 92 | 93.8 | 65.7 | 69.2 | 67.6 |
|  | SVM | 68.2 | 81.5 | 75.5 | 91.3 | 92.0 | 91.7 | 74.3 | 82.1 | 78.4 |
|  | ANN | 72.7 | 77.8 | 79.6 | 95.7 | 96.0 | 93.8 | 80.0 | 92.3 | 81.1 |
| **F_1_ score** | LRA | 0.15 | 0.44 | 0.32 | 0.96 | 0.91 | 0.94 | 0.68 | 0.70 | 0.69 |
|  | SVM | 0.59 | 0.78 | 0.70 | 0.92 | 0.91 | 0.91 | 0.78 | 0.84 | 0.81 |
|  | ANN | 0.70 | 0.79 | 0.80 | 0.96 | 0.96 | 0.94 | 0.85 | 0.94 | 0.85 |
| **n_test_** | | 22 | 27 | 49 | 23 | 25 | 48 | 35 | 39 | 74 |
