# Supplementary figures and images for: Detection of Bacterial Infection in Melon Plants by Classification Methods Based on Imaging Data
Source: Front Plant Sci. 2018 Feb 14;9:164. doi: 10.3389/fpls.2018.00164 (PMC5817087; doi:10.3389/fpls.2018.00164)

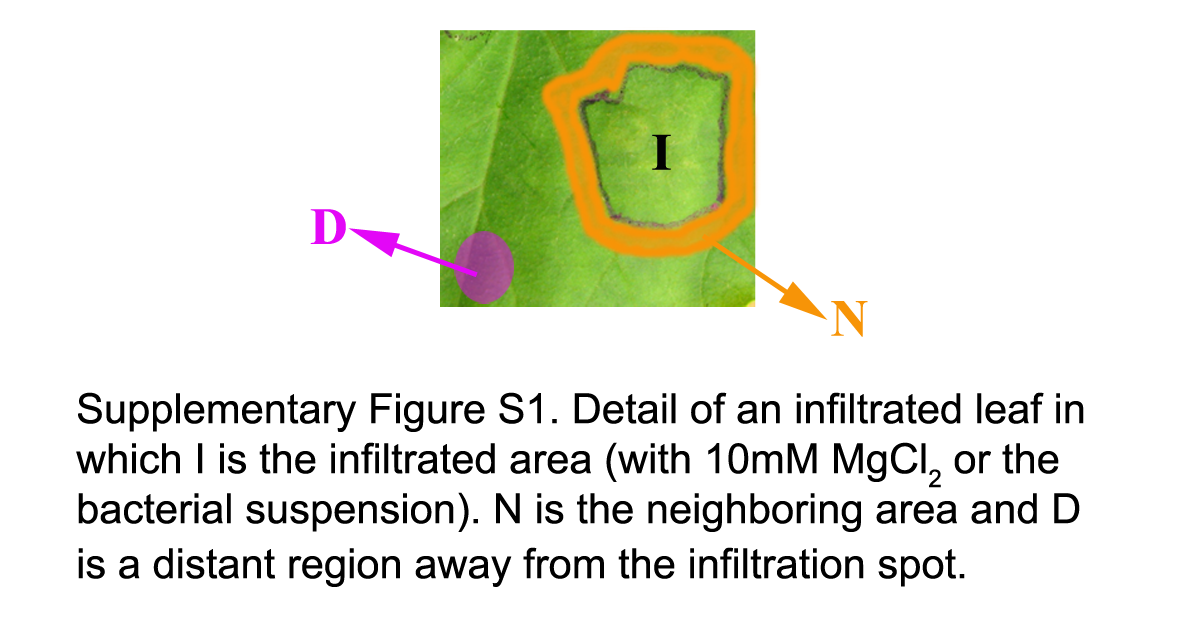

Supplement: Supplementary file 4 [file Image_1.tif]
